# Assessment of human cytomegalovirus co-infection in Egyptian chronic HCV patients

**DOI:** 10.1186/1743-422X-8-343

**Published:** 2011-07-10

**Authors:** Ashraf Tabll, Sahar Shoman, Hussam Ghanem, Mohamed Nabil, Noha G Bader El Din, Mostafa K El Awady

**Affiliations:** 1 Department of Microbial Biotechnology, National Research Center, Giza, Egypt; 2 Department of Microbiology, Faculty of Science, Ain Shams University, Cairo, Egypt

**Keywords:** Hepatitis C virus, Human Cytomegalovirus DNA, Co-infection

## Abstract

Human cytomegalovirus (HCMV) is the most common cause of severe morbidity and mortality in immune- compromised individuals. This study was conducted to determine the incidence of HCMV infection in HCV patients who either spontaneously cleared the virus or progressed to chronic HCV infection. The study included a total of eighty four cases (48 females and 36 males) that were referred to blood banks for blood donation with an age range of 18-64 years (mean age 37.62 ± 10.03 years). Hepatitis C virus RNA and HCMV DNA were detected in sera by RT-nested PCR and nested PCR respectively in all subjects. Immunoglobulin G levels for HCV and HCMV were determined. Besides, IgM antibodies for HCMV infection were also determined in subjects' sera. Fifty three out of 84 cases (63%) were positive for HCV-RNA while 31 (37%) cases had negative HCV RNA. Forty six (87%) and 13 (25%) cases out of 53 HCV RNA positive patients were positive for HCMV IgG and IgM antibodies respectively. While 20 of 53 cases (38%) had detectable HCMV DNA. To examine the role of HCMV infection in HCV spontaneous resolution, two groups of HCV patients, group 1) chronic HCV infection (positive HCV RNA and positive IgG antibodies) vs group 2) spontaneous resolution (negative HCV RNA and positive IgG antibodies) were compared. The percentages of positive CMV IgG and IgM results is higher in chronic HCV patient than those in spontaneously cleared HCV patients and the difference is highly statistically significant (P value < 0.001). Also, there is a general trend towards elevated levels of CMV IgG antibodies in HCV chronic patients than those in spontaneously cleared HCV patients (P value < 0.02). HCMV DNA detection in group 1 was more than twice the value observed in group 2 (38% vs 14.3%, P value < 0.001). Moreover, levels of liver enzymes were significantly higher in HCV RNA positive cases co-infected with HCMV DNA than HCMV negative cases (P value < 0.001). The results indicate the role of HCMV in the liver pathogenesis. We conclude that chronic HCV patients co-infected with HCMV infection can be regarded as high risk groups for liver disease progression where they should be monitored for the long term outcome of the disease.

## Introduction

The importance and the interest of HCMV as a pathogen have increased over the past two decades. Approximately 70-100% of the world's populations are carriers of the virus [1] and it has become the most common cause of severe morbidity and mortality in immune compromised individuals [2]. A primary HCMV infection is followed by a life-long persistence of the virus in a latent state, and reactivation may occur later in life [3]. Therefore, reactivation of the virus is seen during periods of down-regulation of the immune system, such as drug treatment and illness-related stress, or during on-going activation of the immune system such as inflammatory diseases, or co-infection with other pathogens [4]. HCMV can infect virtually all organ tissues, but manifestations of organ involvement generally include symptoms from the liver, the lungs, the intestine and the CNS [5]. Cytomegalovirus is passed from person to person through close contact with body fluids, such as saliva, semen, vaginal fluids, blood, urine, tears and breast milk. Therefore, people can get CMV through sex, breastfeeding, blood transfusions and organ transplants [6]. The cytopathic potential of HCMV in human liver cells was analyzed in cell culture and in tissue sections from patients with HCMV hepatitis, and it was concluded that HCMV can cause direct liver paranchymal damage by efficient cytolytic infection of hepatocytes [6,7]. Human CMV hepatitis occurs as part of disseminated CMV infection. It occurs mainly among liver or kidney transplant recipients or immunosuppressed persons [8,9], however many cases of CMV hepatitis in immunocompetent hosts have also been reported [10,11] so that mild-moderately elevated levels of transaminases and various histopathological changes of the liver were encountered in these patients. On the other hand, HCV is a major health problem in Egypt [12,13]. We have recently shown that co-infection with HCMV could dramatically diminish the possibility of achieving SVR to peg IFN + RBV treatment in chronic HCV patients [14]. This study aims to investigate the incidence of co-infection of HCMV with HCV (either persistent or spontaneously cleared) in samples referred to several blood banks in Egypt.

## Material and methods

### Approval ethics

This research was approved by the Review Board of National Liver Institute, Menoufia University with reference number NLI 0003413 FW0000227

### Study population

Eighty- four subjects who were referred to blood banks in Mansoura city (North Delta of Egypt) for blood donation during the period between February and May 2010 were enrolled in this study. The 84 cases included 48 females and 36 males, with age range 18-54 ± mean of (37.62 ± 10.03) years. Subjects were divided into two groups; patients who were positive for HCV IgG antibodies (*n *= 67) and negative control group (*n *= 17) who were negative for HCV, HIV and HBV antibodies. Informed consents were obtained from each subject before collecting serum and whole blood samples. The age, sex, antibodies against HCV (anti-HCV IgG), antibodies against HCMV (anti-HCMV IgM, anti-HCMV IgG), HCV RNA, HCMV DNA levels, and liver enzyme levels (ALT and AST) were assessed and recorded for both patients and controls.

### Detection of HCV IgG antibodies

Serum samples were collected from all studied subjects to confirm the presence of HCV IgG Abs. HCV IgG Abs were detected using (Diagnostic Automation, INC 23961 Craftsman Road, Suite D/E/F, Calabasas, CA 91302, USA).

### Detection of HCV RNA in all samples

Presence of HCV viremia was confirmed by reverse transcription-PCR using nested primers derived from the highly conserved 5' un-translated region. Serum samples were collected from all cases. Then RNA was extracted from 200 μl of all serum samples using the acid guanidium thiocyanate-phenol-chloroform method [15]. Primer sets used in the detection of HCV RNA were as follow: P1: 5' GGTGCACGGTCTACGAGACCTC 3' - P2 forward primer: 5' AACTACTGTCTTCACGCAGAA 3' - P3 reverse primer: 5' TGCTCATGGTGCACGGTCTA 3'- nested reverse primer P4: 5' ACTCGGCTAGCAGTCTCGCG 3' and nested forward primer P5: 5' GTGCAGCCTCCAGGACCC 3'. All primers were purchased from (Promega, Madison WI, USA). The nested PCR amplification was done in a volume of 50 μl; and the PCR protocol consisted of a reverse transcription step at 37°C for 60 min by using 20 U of cloned Avian Myloblastosis Virus (AMV) reverse transcriptase, 1 × buffer (supplied with the enzyme), (QBIOGENE, USA), 200-400 ng of total cellular RNA as template, 40 units of RNAsin (Clonetech, USA), 0.2 mmol/l from each dNTP (Promega, Madison, Wisconsin, USA) and 10 pmole from primer (P1). First round amplification was done on 10 ul from the first cDNA strand synthesis reaction using 10 pmole from each of (P2) forward primer and (P3) reverse primer, 0.2 mmol/l from each dNTP (Promega, Madison, Wisconsin, USA), 2 units of Taq DNA polymerase (Promega, Madison, Wisconsin, USA) and 1 × buffer supplied with the enzyme. The second round amplification was done similar to the first round, except for using the nested primers (P4) and (P5) as well as 10 ul from the first round PCR product. Thermal protocols for both rounds were 1 min. at 94°C, 1 min at 55°C and 1 min at 72°C for 30 cycles. The products of nested RT-PCR were analyzed on 2% agarose gel electrophoresis.

### Serological analysis of HCMV infection

Human CMV IgM and IgG antibodies were detected in all samples by the qualitative ELISA test using commercially available CMV kits (BioCheck, Foster City, CA, USA). Tests were done according to the manufacturer instructions and results of HCMV IgM and IgG were expressed as O.D. units.

### Detection of HCMV DNA

Total DNA was extracted from 300 μl serum sample using Wizard^® ^DNA purification mini kit, (Promega, Madison, Wisconsin, USA), following the instructions of the manufacturer. Human CMV DNA was amplified using primers derived from the *gB *region of CMV genome and PCR protocols were followed as described previously [16,17]. The amplification mixture contained 3 μl of DNA extract, 10 pmole of each primer gB1: 5' GAGGACAACGAAATCCTGTTGGGCA 3' and gB2: 5' GTCGACGGTGGAGATACTGCTGAGG 3', 0.2 mmol/l from each dNTP (Promega, Madison, Wisconsin, USA), 2 units of Taq DNA polymerase (Promega, Madison, Wisconsin, USA) and 1 × buffer supplied with the enzyme. Two ul from the 1^st ^PCR product were used in a nested-PCR containing the same conditions as mentioned above except for using nested primers gBn1: 5' ACCACCGCACTGAGGAATGTCAG 3' and gBn2: 5' TCAATCATGCGTTTGAAGAGGTA 3'. The thermal cycling protocol was as follows: 1 min. at 94°C, 1 min at 55°C and 1 min at 72°C for 30 cycles. Nested amplification products were visualized on 2% agarose gel electrophoresis and stained with ethidium bromide.

### Liver Enzyme levels

Alanine Amino Transferase (ALT) (normal range, 40 U/L) and Aspartate Amino Transferase (AST) (normal range, 38 U/L) levels were measured in all samples with commercial Kits (Siemens Healthcare Diagnostic Inc., USA) according to the manufacturer instructions.

### Statistical analysis

All statistical analyses were performed using the SPSS 9.0 statistical software program. The statistical significance of difference was considered when p ≤ 0.05.

## Results

### Prevalence of HCV IgG Abs and HCV RNA in studied subjects

**Figure 1:**
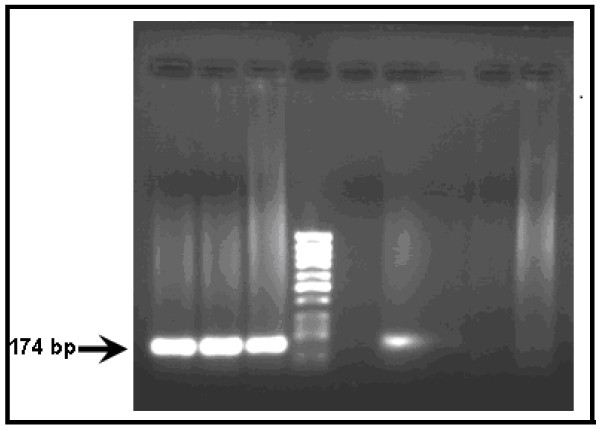
**RT nested PCR results of serum samples lane 1, 2, 3 and 6 were positive for HCV RNA while lanes 5, 7, 8 and 9 were negative for HCV**.

The results of HCV IgG Abs showed that 67 patients were positive for HCV IgG Abs while 17 were negative for HCV IgG Abs. The presence of HCV viremia was confirmed by RT-nested PCR. The RT-PCR products of HCV RNA in some subjects are shown in Figure 1. A result was considered positive when a clear 174-bp product was visible on agarose gel stained with ethidium bromide. In the sixty-seven patients who were positive for HCV IgG Abs, HCV RNA was detected only in fifty three subjects.

Patients were classified according to the results of HCV IgG Abs and HCV RNA as follows: fifty three patients (31 females and 22 males) were positive for (both HCV RNA and HCV Abs) and they are described as group (1), while 31 cases (17 females and 14 males) were negative for HCV RNA. Among the later category (negative HCV RNA) 14 cases had detectable HCV IgG Abs (negative HCV RNA, positive HCV Abs) and were referred to as group (2) while 17 cases were negative for both HCV RNA and IgG Abs), and they are described as group (3).

### Prevalence of HCMV Abs in chronic HCV patients versus controls

**Figure 2:**
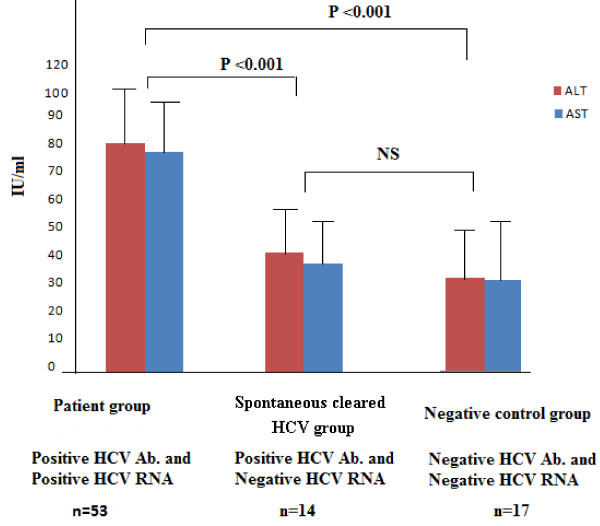
**Measurement of liver function tests (ALT and AST) in different studies groups**. **ALT: **Alanine Amino Transferase. **AST: **Aspartate Amino Transferase. **NS: **Non Significance

To investigate whether the prevalence of HCMV Abs (IgG and IgM) is higher in chronic HCV patients (group 1) than in comparable control subjects (groups 2 and 3), titers of both classes of immunoglobulins were measured in all subjects groups. Thirteen (25%) and 46 (87%) out of 53 chronic HCV patients (group 1) had detectable IgM and IgG HCMV Abs respectively. No gender preference was noted (6/13 males, 7/13 females were positive for HCMV IgM and 21/46 males, 25/46 females were positive for HCMV IgG). In group 2 (negative HCV RNA positive Abs) 2/14 (14.3%) and 11/14 (78.6%) were positive for HCMV IgM and IgG respectively. While in group 3 (negative for both HCV RNA and Abs), 3/17 (17.6%) and 9/17 (53%) were positive for IgM and IgG HCMV Abs respectively. The results depicted in Figure 2 showed that percentage of positive CMV IgG and IgM is higher in chronic HCV patients than those in spontaneously cleared HCV patients and the difference is highly statistically significant (P value < 0.001). Results showed that 25% of HCV patients had IgM vs 14.3% in controls (group 3) while 87% in chronic HCV patients had IgG HCMV Abs vs 78.6% in spontaneously cleared HCV patients (group 2). Also, there is a general trend towards elevated levels of CMV IgG antibodies in chronic HCV patients (0.973 ± 0.61) than those in spontaneously cleared HCV patients (0.625 ± 0.42), P value < 0.02.

### Prevalence of HCMV DNA in chronic HCV patients versus controls

**Figure 3:**
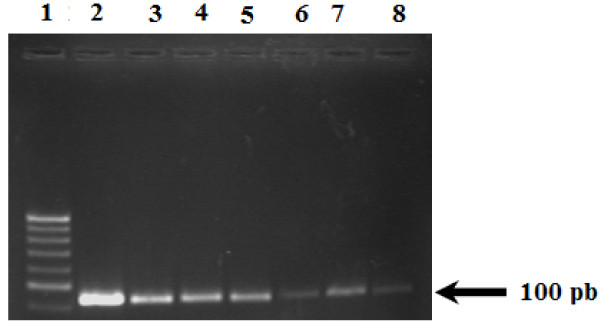
**Nested PCR results of HCMV DNA in serum samples**.

**Figure 4:**
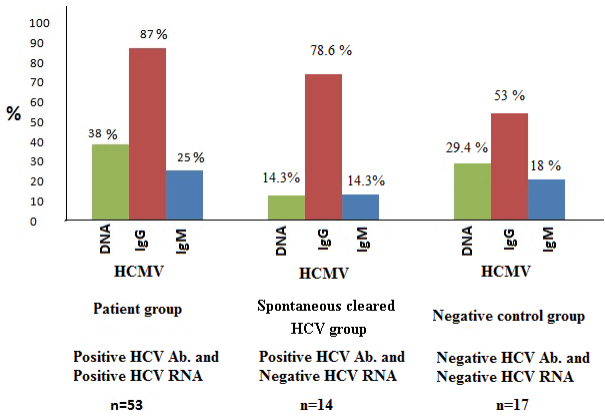
**Detection of HCMV DNA, HCMV IgG and HCMV IgM antibodies the studied groups**.

The nested PCR product of CMV gB gene in some subjects is shown in Figure 3. A result was considered positive when a clear 100-bp product was visible on agarose gel stained with ethidium bromide. The results displayed in Figure 4 demonstrated that 20/53 (38%) chronic HCV patients had detectable HCMV DNA in their sera, compared with 2/14 (14.3%) of HCMV DNA positivity in group 2 (spontaneously cleared HCV infection group, negative RNA, positive Ab) and 5/17 (29.4%) in group 3 (control subjects, negative for both HCV RNA and Abs). Of the twenty positive HCMV DNA in chronic HCV cases 17 (85%) had detectable HCMV IgG while 13 (65%) only had detectable IgM with no gender preference. The observed difference between HCMV DNA prevalence in chronic HCV patients (38%) and spontaneously cleared HCV patients (14.3%) was highly statistically significant (P < 0.001).

### Levels of ALT & AST among study groups

**Figure 5:**
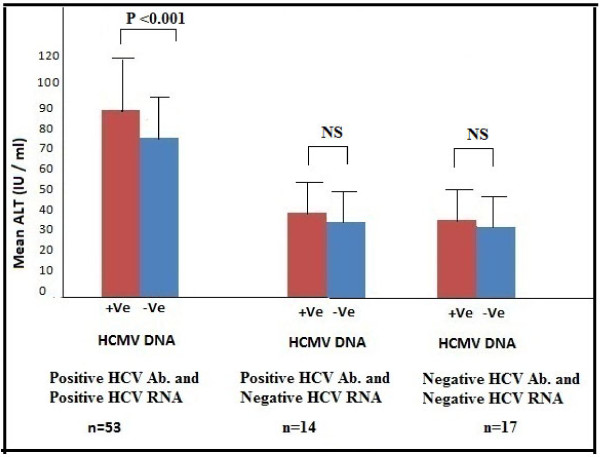
**Measurement of ALT in patients positive and negative for HCMV DNA in the different studied groups**. **ALT: **Alanine Amino Transferase. **+ Ve: **Positive. **-Ve: **Negative. **NS: **Non Significance

The results presented in Figure 2 clearly demonstrated a gradual decline in both ALT and AST levels from group 1 to group 2 and group 3. In HCV persistent group, serum ALT levels (mean: 82.7 ± 20.6 IU/L) and AST (mean: 79.2 ± 21.7 IU/L). In spontaneously cleared group serum ALT mean levels were 42.9 ± 9.8 IU/L) and AST were 39.4 ± 10.75 IU/L) while in negative control group 3 where serum ALT mean levels were 33.6 ± 10.7 IU/L and AST mean levels were 32.6 ± 11.4 IU/L). Serum ALT and AST levels were significantly higher in group 1 than in group 2 and group 3 (P value < 0.001) with no significant difference between groups 2 and 3. Interestingly, in HCMV-positive chronic HCV patients, both ALT (mean: 93.2 ± 24.9 IU/L) and AST levels (mean: 90.2 ± 24.6 IU/L) were higher than that of HCMV-negative chronic HCV patients where mean ALT values were 71.85 ± 15.1 IU/L) and AST were 70.5 ± 16.9 IU/L). The difference was highly statistically significant (P value < 0.001) as shown in Figure 5.

## Discussion

Cytomegalovirus is a ubiquitous b-herpes virus that affects 60-80% of the human population. Infection with CMV is more widespread in developing countries. In general, HCMV infections are effectively controlled by the immune system, but without the ultimate clearance of the virus. Instead, the viral genome is retained at specific sites in a latent state out of which reactivation to recurrent infection can occur [18]. The diagnosis of active CMV infection was based on the detection of CMV replication in the blood. Therefore reactivation of CMV in the absence of an effective immune response is central to the pathogenesis of the disease [2]. In this study, we investigated the incidence of HCMV infection in Egyptian HCV patients who either spontaneously cleared the virus or progressed to chronic HCV infection and we examined the potential role that CMV plays in HCV progression. The present data showed that the percent of positive HCMV Abs were significantly higher (P < 0.001) in chronic HCV patient than those in spontaneously cleared HCV patients. Also, the CMV DNA was detected in 38% of chronic HCV infected patients (65% of them had positive IgM antibodies) compared with 2 out of 14 (14.3%) spontaneously cleared HCV patients (where both patients had positive IgM antibodies). The percentage of positive CMV DNA results is higher (P value < 0.001) in chronic HCV patients compared to spontaneously cleared HCV patients, thus indicating a significant association between HCV progression rate and CMV reactivation. Moreover, the results confirmed that the detection of CMV DNA by PCR in peripheral blood leukocytes is a sensitive and reproducible procedure for detecting viral infection. As the serological methods reported to be insensitive and can't distinguish between CMV infection and CMV disease as IgM antibodies may persist for months or years and may be detected during reactivation of latent virus infections [19]. Recently, DaPalma et al., [20] classified virus-virus interactions by organizing them into three main categories: (1) direct interactions of viral genes or gene products, (2) indirect interactions that result from alterations in the host environment, and (3) immunological interactions, unique to organisms equipped with an adaptive immune system. In the present study the reason (s) why both HCMV IgM (17.6% vs. 14.3%) and CMV DNA (29.4% vs. 14.3%) were more frequently detected in healthy controls than in spontaneously cleared HCV subjects are not clear. However, we may suggest that virus-induced changes that may affect co-infecting viruses involve the innate immune mechanism induced by type I interferon known as the antiviral state. The antiviral state consists of increased expression of a combination of enzymes, which if activated, shut down cellular translation [21,22]. The most critical of these enzymes are PKR and 2'-5'OAS. In negative control group, CMV will replicate solely without the involvement of another virus i.e. HCV which explains the higher prevalence of CMV DNA in controls than spontaneously cleared HCV patients. In earlier studies on dual viral infections (HBV/CMV and HCV/CMV) [6,23] it was demonstrated that CMV was detected in HBV or HCV patients mostly as a dual infection and that it can aggravate the course of the disease. Recently, Bader El din et al., [14] reported higher rate of CMV co-infection in chronic HCV Egyptian patients than those reported in other patient populations. Whether HCV predisposes patients to CMV infection or CMV predisposes patients to HCV is not clear. Besides, Lian *et al*., [24] reported a high mortality rate (85.7%) in CMV and HBV co-infected patients compared to HBV infected patients only. Different studies reported that CMV causes hepatitis with inflammation and fibrosis of liver cells. That means CMV affects the liver and overall immunological status of the host body [6,14,25]. Bayram et al., [6] reported elevated liver enzymes and marked histological changes in the liver of HCMV-HCV co-infected patients. The serum levels of ALT and AST enzymes showed a highly significant (P value < 0.001) elevation in positive HCMV DNA than in negative subjects, thus suggesting a role of HCMV in liver pathogenesis and support the results of Razonable et al., [26]. Furthermore, Rafael et al., [27] reported that HCMV infection interacted with HCV and raised the influence on the liver enzymes and cause hepatitis. Considering the fact that HCMV viruses exert an immunomodulatory effect resulting in enhanced immunosuppression [28,29] and cytokine dysregulation which could accelerate HCV pathogenesis in critically ill patients, the findings of the present study support the hypothesis that even low level of HCMV replication doesn't evolve into clinical disease it significantly influences HCV outcome.

## Competing interests

The authors declare that they have no competing interests

## Authors' contributions

AT designed the study, wrote the final version of the manuscript. SS followed up all technical steps. HG followed up all technical steps. MN participated in extraction of DNA and RNA and PCR for HCV RNA and HCMV DNA. NB participated in writing the draft. ME finalized the manuscript in its final form. All authors read and approved the final manuscript
